# Efficacy of baby-CIMT: study protocol for a randomised controlled trial on infants below age 12 months, with clinical signs of unilateral CP

**DOI:** 10.1186/1471-2431-14-141

**Published:** 2014-06-05

**Authors:** Ann-Christin Eliasson, Lena Sjöstrand, Linda Ek, Lena Krumlinde-Sundholm, Kristina Tedroff

**Affiliations:** 1 Neuropediatric Unit, Department of Women’s and Children’s Health, Karolinska Institutet, Stockholm, Sweden

**Keywords:** Constraint-induced movement therapy, Cerebral Palsy, Upper limb, Hand function, Early intervention

## Abstract

**Background:**

Infants with unilateral brain lesions are at high risk of developing unilateral cerebral palsy (CP). Given the great plasticity of the young brain, possible interventions for infants at risk of unilateral CP deserve exploration. Constraint-induced movement therapy (CIMT) is known to be effective for older children with unilateral CP but is not systematically used for infants. The development of CIMT for infants (baby-CIMT) is described here, as is the methodology of an RCT comparing the effects on manual ability development of baby-CIMT versus baby-massage. The main hypothesis is that infants receiving baby-CIMT will develop manual ability in the involved hand faster than will infants receiving baby-massage in the first year of life.

**Method and design:**

The study will be a randomised, controlled, prospective parallel-group trial. Invited infants will be to be randomised to either the baby-CIMT or the baby-massage group if they: 1) are at risk of developing unilateral CP due to a known neonatal event affecting the brain or 2) have been referred to Astrid Lindgren Children’s Hospital due to asymmetric hand function. The inclusion criteria are age 3–8 months and established asymmetric hand use. Infants in both groups will receive two 6-weeks training periods separated by a 6-week pause, for 12 weeks in total of treatment. The primary outcome measure will be the new Hand Assessment for Infants (HAI) for evaluating manual ability. In addition, the Parenting Sense of Competence scale and Alberta Infant Motor Scale will be used. Clinical neuroimaging will be utilized to characterise the brain lesion type. To compare outcomes between treatment groups generalised linear models will be used.

**Discussion:**

The model of early intensive intervention for hand function, baby-CIMT evaluated by the Hand Assessment for Infants (HAI) will have the potential to significantly increase our understanding of how early intervention of upper limb function in infants at risk of developing unilateral CP can be performed and measured.

**Trial registration:**

SFO-V4072/2012, 05/22/2013

## Background

Recent knowledge of the great plasticity of the young brain indicates that it is important to start training at an early age. There have so far been no early-intervention programmes designed to improve hand function in infants with cerebral palsy (CP); most programmes have instead targeted general motor and cognitive development [1,2]. Existing studies of early intervention mainly cover preterm infants, sometimes even excluding children with CP because of its heterogeneity. One reason for the lack of established intervention programmes for hand use in infants, is uncertain diagnosis. The most accurate predictive tool for CP is brain imaging combined with Prechtl’s Assessment of General Movements administered up to 4 months post term [3-5]. However, many children with unilateral CP are born at term with no adverse birth events. Whether or not there is a suggestive neonatal history, obvious signs of unilateral CP usually do not appear until 4–5 months of age. Unilateral CP is a common subtype of CP and brain imaging reveals that white-matter lesions and cortical/subcortical lesions are the commonest types of brain lesions [6]. However, brain lesions do not necessarily result in CP; for example, only approximately 30% of all children with neonatal stroke will eventually develop unilateral CP [7], and even haemorrhages and other incidents in preterm children do not necessarily lead to CP [5]. This makes it difficult to decide which children might benefit from early-intervention programmes.

In recent decades it has become clear that hand function can be improved by active motor training in older children [8,9]. Modified constraint-induced movement therapy (CIMT), an effective method for such training, is based on constraint of the non-involved hand and intensive activity-based training of the impaired hand. Before CIMT should be used for infants, however, it needs to be tested for feasibility and its dosage and degree of restraint adjusted to suit young infants. More importantly, no available assessments describe signs of or monitor hand use development in such young infants. This paper describes a special adjustment of the intervention method, baby-CIMT, to be used in an RCT evaluated using our newly developed Hand Assessment for Infants (HAI) to measure hand function in each hand separately and both hands together.

### Translation of learning-induced brain plasticity into clinical practice

Early intervention is expected to be important, as neural networks and pathways that remain intact after brain injury can be strengthened through learning-induced plasticity. After Hubel and Wiesel’s ground-breaking discoveries of visual system plasticity in the 1970s, Nudo et al. [10] demonstrated that neural activity induces synaptic changes in the sensory and motor cortex. The corticospinal system also experiences ongoing structural change [11-13]. More recent animal studies have demonstrated that there is a critical period of motor system plasticity, and that activity-dependent reorganisation of the motor-projection pattern to the hand occurs before about 1 year of age [14,15]. Activity-based training using a model of CIMT synchronised with the development of the corticospinal tract in an animal model of CP restored motor function and induced structural changes in the corticospinal system [16,17]; this restoration was not found if the intervention was implemented at a higher age. Increased knowledge of early brain plasticity supports increased interest in early intervention and its impact on the development of the hand motor system. In this project, we would like to exploit the great plasticity of the young brain, hypothesising that treatment at an early age will influence the future level of development. This hypothesis is based on the assumption that there are critical periods during human brain development in which treatments are more effective than they would be later on.

### Object exploration and manipulation in the first year of life

To evaluate any hand training programme for infants, it is important to have a good understanding of the natural history of hand development in young children. Voluntary action starts to emerge at birth, and von Hofsten [18] has demonstrated that reaching and grasping actions can be detected even in newborns, though it takes some months before grasping actions are obvious and frequent. In the first year of life, infants gradually gain remarkable control over their hands, exploring and manipulating objects with increasing skill [19]. Infants consciously explore objects, typically using the hand nearest an object or both hands together [20]. There is typically no asymmetry or hand preference before about 9 months of age in typically developing children [21], nor is coordinated bimanual hand use seen until about 8 months of age, when infants start performing more complex sequences, such as removing a lid to grasp a toy [22]. Typically developing infants use both hands equally.

### Early hand motor function in children with unilateral CP

The development of hand use below the age of 12 months in infants with unilateral CP has not been described. It is not known when deviation from normal development can first be detected nor what the typical signs of unilateral CP might be. Textbooks usually describe unilateral CP as characterized by a flexed elbow, pronated forearm, and thumb in the palm without applying a developmental perspective. We have recently started to monitor infants from early ages who are likely to develop unilateral CP and it is clear that the amount and quality of hand use develops differently in the two hands at an early age. Asymmetric hand use can already be seen at the age of 3–5 months. Using our newly developed instrument, HAI for infants aged 3–12 months, we can for the first time describe and measure this difference from typical early motor development (Krumlinde-Sundholm et al., in preparation). HAI measures each hand separately in uni- and bimanual play-tasks. This means that HAI will fill an important gap, because no currently available outcome measure can quantify the development of asymmetric hand function at such a young age, and available norm-referenced tests do not reflect the deviant development seen in children with unilateral CP [23,24]. Based on our ongoing research, we know that variation among infants is considerable and that developmental differences range from negligible to dramatic during the first year of life.

### Theoretical assumption for intervention

Two types of interventions based on different theoretical assumptions will be tested in this project. Briefly stated, the CIMT programme for infants (baby-CIMT) is based on self-initiated action and assumes that one must practice the motor action one intends to learn. The baby-massage intervention is based on the assumption that general tactile stimulation is important for development. Both interventions assume that early stimulation is important; by using baby-massage as a comparison group, we can control for the placebo effect of increased attention and time spent with the child. The theoretical assumptions and evidence supporting both methods will be described in greater depth.

#### Baby-CIMT

CIMT is characterized by restraint of the well-functioning upper limb (irrespective of restraint device/type) and intensive structured training (irrespective of training type) [25]. CIMT needs further development and adjustment to be appropriate and feasible for infants. Baby-CIMT has been developed in our research group and its modification will be described for the first time here. Baby-CIMT is a manual motor training programme shaped by several important perspectives on infant development with the general aim to increase the amount and quality of hand use.

The assumption underlying baby-CIMT, as in the case of the original CIMT model, is that the practice of self-initiated motor actions is crucial for motor development. The theoretical perspectives that have shaped the baby-CIMT programme are pretty similar to those that shaped the eco-CIMT programme for children above 2 years of age [26]. The baby-CIMT play session, i.e., the active motor training, is influenced by dynamic systems theory, a model highlighting the importance of children’s self-initiated activity [27]. From this perspective, we assume that development is driven by children’s unique characteristics and capacity to explore the environment, through which they discover new abilities. This model also emphasises the importance of a rich immediate environment in which a varied selection of toys and other objects facilitates the development process. The principles of motor learning, i.e., how individuals acquire and perform motor activities [28], also underpin baby-CIMT. To promote motor activity in infants, selecting appropriate toys and play objects at just the right ability level is crucial. Repetition is also key, as is feedback on performed behaviour.

Bronfenbrenner and Morris’s ecological model of child development highlights the interaction between the active child and other people, objects, and symbols in the immediate environment [29], suggesting that baby-CIMT must be child centred. Bronfenbrenner and Morris further state that unconditional love and time spent with a child are the two most important agents driving development. This approach, and that of family-centred service (FCS), will guide the present intervention. FCS comprises a set of values, attitudes, and approaches towards the family [30]. When coaching and guiding parents to be the training providers, motivational interviewing [31] and solution-focused coaching [32] are important techniques used by therapists. By using these techniques, therapists can help increase parents’ motivation to be treatment providers and empower them to develop goals and implement the programme. Simply stated, the therapist should ask questions rather than come up with answers; in particular, the therapist must express empathy and build a relationship with the parents. Education will also be part of the programme, and parents’ stress can decrease after they read about and understand the individuality and needs of their infants [33]. Parental education about the infant’s situation is known to enhance cognitive and social function in the infant [34].

#### Baby-massage

Massage is defined as systematic touch by human hands, consisting of gentle, slow stroking of each part of the body in turn. It is often combined with other forms of stimulation, such as kinaesthetic stimulation (e.g., passive extension/flexion of the arms and legs), talking, and eye contact [35]. The overall assumption underlying the baby-massage intervention is that tactile stimulation promotes overall development. The techniques and dosage used vary considerably. Baby-massage is assumed to affect physical health and growth, and factors such as weight gain and body length are expected to be influenced by massage, as are crying and sleeping/waking behaviour [35]. Baby-massage is used for typically developing children in several cultures, and today there is increasing interest in it among parents in western cultures. It is of special interest in neonatal intensive care units (NICU), where the environment can be a stress factor and tactile input can be lacking [36]. There are indications that baby-massage can improve the development of gross and fine motor skills as well as psychomotor development in preterm children [37], a possibility supported by a recent Cochrane review [35]. Children suffering from early brain lesions have been treated with baby-massage in very few studies, one of which found reduced muscle tone and improved fine and gross motor function [38]. It is well known that parent–infant interaction is dependent on parental ability to respond appropriately to the infant’s emotional state. In the fields of developmental psychology and infant mental health, baby-massage is expected to support early parenting and strengthen parent–infant communication [39]. Various mechanisms have been proposed for how massage might benefit infants. The biological rationale for using massage to improve growth and development in preterm or at-risk infants is that it may increase metabolic efficiency while reducing stress behaviour or the production of stress hormones [35]. Baby-massage will be used as an intervention in comparison with baby-CIMT based on its possible effect on motor development but also for its positive effect on parent–infant interaction. Evidence of the long-term benefits of all aspects of baby-massage is still weak, and it would be useful to explore its effects in high-risk infants [35].

## Methods and design

This paper describes the methodology of an RCT comparing the effects of the baby-CIMT and baby-massage protocols on the development of manual ability in infants at risk of developing unilateral CP. The two treatment protocols will be described in detail. HAI was selected as the primary outcome measure with the main aim of measuring the development of manual ability in both hands.

### Ethical considerations

The study has been approved by the Stockholm Regional Ethical Review Board (no. 2009/1100-32). All parents will be given oral and written information about the study before being asked to sign an informed consent form. The randomisation will be performed after the form is signed.

### Primary objective

The primary objective of the study is to investigate the effects of baby-CIMT and baby-massage on the development of manual ability in the first year of life of infants at risk of developing unilateral CP.

The specific hypotheses to be tested are:

1. Baby-CIMT is a feasible method for families and infants below one year of age.

2. Infants receiving baby-CIMT will develop manual ability in the involved hand faster than will infants receiving baby-massage in the first year of life.

3. Improvement of manual ability in the involved hand will be faster during the training period than during a period without training in the baby-CIMT group.

4. The manual development of the involved hand will depend on the type of brain lesion. Infants born at term with neonatal stroke are expected to develop more slowly than will preterm infants with mainly white matter lesions, independent of group allocation.

5. Development of manual ability in the non-involved hands will not differ between groups.

6. The assumed difference in manual development in the involved hand at 1 year of age depends on group allocation and the difference will remain at 2 years of age.

The secondary objective is to investigate whether the different treatment protocols influence the parents’ self-rated parenting competence.

1. Parents in the baby-CIMT programme will feel more competent at parenting than will parents in the baby-massage group since they will have learned more about the child’s specific needs.

### Trial design

**Figure 1:**
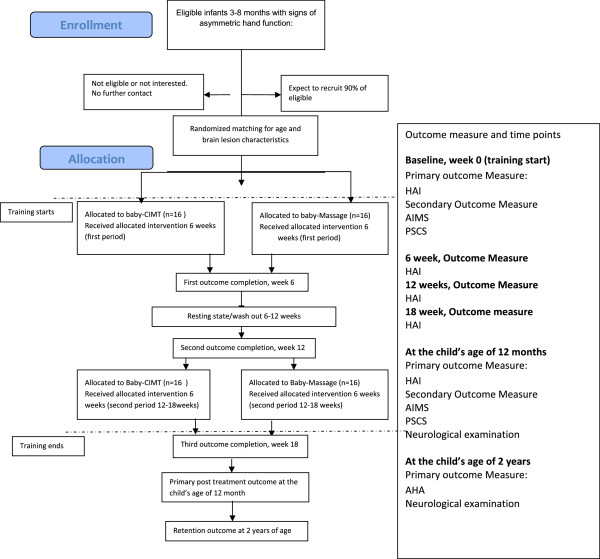
Flowchart of a baby-CIMT trial according to CONSORT guidelines.

The study will be a randomised, controlled, evaluator-blinded prospective parallel-group trial based on the Consolidated Standards of Reporting Trials (CONSORT) statement regarding the randomised trial of non-pharmacological treatments [40]. There will be two arms, baby-CIMT and baby-massage; children randomised to either arm will receive two 6-week training periods separated by a 6-week break (Figure 1). The study setting is Astrid Lindgren Children’s Hospital, a tertiary hospital in Stockholm, Sweden.

#### Recruitment of children at risk of developing unilateral CP

Two groups of children will be invited: 1) infants at risk of developing unilateral CP due to a known neonatal event affecting the brain and 2) infants referred to Astrid Lindgren Children’s Hospital due to asymmetric hand function. A known etiological cause of the asymmetric hand use is not a prerequisite for referral, i.e., infants with neurological signs but without a diagnosis can be referred to the project. Recruitment (different from inclusion) is based on broad inclusion criteria in order not to overlook children who may later develop unilateral CP; the diagnosis will be followed up at a higher age. Signs of asymmetric hand use will be confirmed using the Hand Assessment for Infants (HAI). If clinical signs of asymmetry are inconclusive at referral, a second investigation will be performed one month later. Neuropediatricians and neonatologists in Stockholm-area hospitals will be informed of the study through seminars and hand-outs.

#### Participants

Infants are eligible to enter the study based on the following inclusion criteria: 1) 3–8 months of age, corrected age (CA) being used for preterm infants, and 2) clinical signs of asymmetric hand use, confirmed by the asymmetry score on the primary outcome measure HAI. Exclusion criteria will be severe visual impairment, seizures not controlled by antiepileptic drugs, and children with clinical signs of bilateral involvement.

Inclusion at 3–4 months of age is based on typical infant development of self-initiated actions. Before this age, self-initiated actions are difficult to measure and hand asymmetries difficult to detect using HAI. Information from HAI video-recordings indicates that, at this age, infants begin to be interested in the test toys; at 3–4 months, the children’s grasping ability is still limited but their interest in handling toys is increasing, making it possible to initiate baby-CIMT. Children will be included no later than at 8 months of age because the protocol lasts 18 weeks and the intervention is intended to finish before the children are 1 year old.

At 1 year of age, or 1 year CA, the children will be examined by a paediatric neurologist. Children who meet the Surveillance for Cerebral Palsy in Europe (SCPE) criteria at this time will be diagnosed as likely having unilateral CP. The diagnosis will be based on a neurological examination and thorough history, including aspects of fine and gross motor development and information regarding neonatal events. Preterm birth, neonatal stroke, asphyxia, or other incidents such as meningitis or insults after early heart surgery will be considered. If known, we will also consider brain imaging results indicating conditions such as predominantly unilateral haemorrhage or early signs of white matter damage of immaturity (WMDI), neonatal stroke, and malformations. The CP diagnosis and subtype will be updated as other symptoms may appear later.

#### Randomisation

Eligible infants will be randomly assigned to the baby-massage or baby-CIMT groups in a block design. The infants will be stratified by age and neonatal events, with three age groups, i.e., 3–4, 5–6, and 7–8 months, and three neonatal event groups, i.e., neonatal stroke in full-term infants (> week 37), preterm birth (< week 37), and unknown/other. This stratification is chosen to ensure that the study groups will be approximately equally distributed based on age and neonatal events.

Infants will be recruited consecutively. Randomisation will occur after the first assessment, ensuring that the assessing occupational therapist will not be biased at this time by knowing the group assignment. A random number list that also contains stratification alternatives will be generated before the start of the study and kept by the Principal Investigator (PI) in a locked room. When an infant is recruited, relevant stratification information will be given to the PI; the infant will then be assigned to the next position and added to the list by the PI. If an infant is later excluded for any reason, the infant’s position in the randomisation list will not be replaced by any new infants.

#### Blinding

Families will not be blinded to group allocation, though they will be blinded to the study hypotheses. The occupational therapist responsible for data collection (i.e., administration and filming of HAI and AHA) will not be blinded to group allocation. However, the occupational therapist scoring the video recordings of HAI and AHA will be blinded to group allocation. The physiotherapist administering the Alberta Infant Motor Scale will not be otherwise involved in the study (see “Outcome measure and procedure”).

### Sample size

The estimated number of participants needed to achieve the study objectives is based on HAI data obtained from a pilot group of 12-month-old infants who received either studied intervention. Based on a two-tailed test of two independent means, with a significance (α) level of 0.05 and 80% power, we require 16 participants in each group for a total sample of 32.

### Study protocol

The time schedule for enrolment, interventions (including any run-ins and washouts), assessments, and participant visits is presented schematically in Figure 1. Data collection will occur at the start of intervention, during the study period after 6, 12, and 18 weeks, and post intervention and when children are 1 and 2 years of age (Figure 1).

Both groups will receive two 6-week intervention periods separated by a 6-week pause. The 6-week pause (washout) was chosen to allow the development of hand use to be monitored without any structured intervention in both groups. The intervention frequency will be 6 days/week in both groups. For baby-CIMT, the training duration will be 30 minutes per day for a total dosage of 36 hours. For the baby-massage group, the massage will last 5–30 minutes daily depending on infant interest (see description of protocol in “Baby-massage”). Every day, the treatment duration will be recorded in a diary. Parents will be trained to provide the type of treatment their infant has been assigned. Data collection and neurological examination will occur in the hospital. Instruction in baby-massage will be given at the hospital. Baby-CIMT implementation will be monitored through home visits. Physiotherapy or other interventions will continue as usual for both groups.

Project organisation is handled mainly by the PI (ACE). Reviews of the project process for both cohorts will be planned regularly by the investigation team. Data collection, i.e., video recording of HAI testing, will be done by the occupational therapist (LS), who is also responsible for training and education in the baby-CIMT group. A certified baby-massage instructor is responsible for training and education in the baby-massage group.

#### Baby-CIMT

This programme will be based on experience from a pilot group of infants, previous work with children older than 18 months, and the theoretical considerations described in the introduction. The reasons for choosing the training dosage and type of restraint will be reported and various programme components further described.

##### Restraint type and training dosage

Any kind of simple restraint can be used on the non-involved hand. The restraint is only used during the training. From the pilot group we learned that young infants will rarely remove the restraint but will commonly start to use the involved hand as soon as the non-involved hand is rendered less useful by the imposition of a soft restraint. We prefer to keep the restraint simple and comfortable, so a sock or mitt can be used as well as a bag clip at the end of a long-sleeved sweater.

The total dosage will be 36 hours, administered over the two 6-week training periods. The most effective combination of training hours/day and length of training period is currently unknown even for older children [25]. In the present study, we have chosen 30 minutes per day of training based on practical considerations and feasibility. First, the attention span of infants is short: even 30 minutes can be too long and may sometimes need to be divided into two shorter sessions. Second, limited time is available when young infants are alert and awake and this time needs to be shared with other caring needs. To obtain a reasonably high dosage, the length of the programme will be 12 weeks. Parents like to divide the time into two 6-week periods; it gives them time to focus on other things between the periods of treatment and it means that the overall treatment time frame is longer within the first year of life. This longer period takes advantage of the children’s developmental progression in relation to handling toys. Concern has been raised that restraining the non-involved hand may negatively affect its development. This concern will be addressed by means of the short duration of daily restraint and the infants’ continued use of both hands outside the treatment time. We also assume that the infants will immediately start to apply what they learned in the training session at other times of the day. By using HAI as an outcome measure, it will also be possible to monitor the development of each hand separately.

##### Components of baby-CIMT programme

Baby-CIMT includes several components in which training in grasping action and toy exploration is the main focus. However, the training will not be effective if it is not integrated with the other theoretical assumptions, including the expectations and attitudes towards the infant and families (see “Background”). The need for a rich environment, selection of toys at the right ability level, optimal child positioning, and the education and supervision of the parents as treatment providers are important components of the baby-CIMT intervention.

##### Attitudes towards the infant

Both therapists and parents must be aware of and recognise the infants’ response pattern and their intention to grasp and explore objects as well as interact and communicate. In general, young infants react slowly and, compared with older children, they take considerable time to initiate motor actions; infants with an affected hand are even slower to initiate actions. Some key advice is to: a) wait for the child’s intention while holding his or her attention; b) attract the child’s attention and encourage him/her to act, but without forcing; c) reinforce and respond positively to the child’s actions; and d) stop the training when the child becomes tired and stops cooperating.

##### Toy selection for training dependent on infant’s ability

Toy selection is vital for the ability to practise various hand actions. Importantly, toys can be any play objects of interest. When choosing a toy, the infant’s age, cognitive level, and motor ability must be taken into consideration. It is important to choose toys and expect motor actions to the appropriate ability level. Young infants or infants with limited motor ability need at first to learn to initiate reaching toward objects. Toys needs to be within their reaching distance to stimulate touching and moving the objects and certain pre-grasping behaviors. To stimulate grasping, easily grasped toys must be presented near the hand that is expected to grasp. When the manual ability of infants become more advanced, they will develop their grasping ability and become interested in exploring objects. At this ability level, infants like to have a lot of toys to explore and numerous grasping actions can easily be promoted. Infants typically explore and manipulate toys and other play objects by banging, fingering, mouthing, slapping, and dropping them. Thereafter, the infants have to refine the quality of grasping and object manipulation. This means that one must give infants a series of small objects to pick up to stimulate precise grasping, well-adapted to the objects’ properties. Importantly, the infants have to continue to practice on their ability level. The challenge for therapists is to choose toys and play situation on an appropriate ability level, not to difficult not to easy. Result on the HAI can guide the decisions.

Families are advised to collect toys and play objects for the training sessions in a special basket; these toys should be used only during the training sessions. It is known that new things are more attractive than familiar things and that even young infants are more likely to look at and manipulate novel toys than familiar toys [41]. There should be many toys in the basket in order to promote repetition. To elicit the different motor actions the toys also needs to have different characteristics. Play objects must be of appropriate size and weight according to the infant’s grasping ability. Likewise, the toys have to be made of various materials ensuring the infant interest in object exploration. Many objects in the environment are interesting to infants, including necklaces, kitchen utensils, Christmas decorations, soft packages, and natural items such as stones and pinecones. As the infants become older, they must be presented with new toys matching their cognitive level.

##### Position of infants and parents during hand training

The parents should always be positioned in front of the infant to facilitate interaction and allow the parents to easily see the infant’s reaction to the toys. In general, the infant should be sitting in as upright and stable position as possible to facilitate self-initiated actions [42]. Before they are able to sit, infants can be placed in a baby seat/bouncer; when the infants can sit in a high chair, this is preferable. If the infant is somewhat unstable, small pillows should be used to provide stability: we do not want the infant to have to concentrate on maintaining sitting balance when trying to use his or her hand. When an ordinary table is used, it should not be too high. When the infant is able to sit independently, he or she can of course be on the floor. The prone position should be avoided for hand training, we do not want them to practice weight bearing concurrently with object manipulation.

##### Therapist’s role and attitudes towards the family

It is known that parents can be effective treatment providers if they are properly trained and supervised [25,43]. To ensure high-quality training, participating families will receive supervision, coaching, and education during weekly home visits from an occupational therapist. The attitude of the therapist is crucial to training success, as the therapist must: a) support the parents’ sense of self-efficacy and confidence as treatment providers, so they can make the treatment situation enjoyable; b) empower the parents as problem solvers and experts regarding their child’s ability, as people who can use their creativity to find suitable toys for practice; c) ask questions that are open-ended and thus help parents come up with their own answers; and d) summarise what is discussed in the training sessions.

##### Information material and parent education

Parents will be given a folder of material presenting the various aspects of the programme from a family perspective. It includes a diary for recording training times/durations, notes for focus areas, and mind maps of important questions to consider before and after the daily training. The folder will also include suggestion of play material and written information addressing particular concerns and interests. Questions to be covered in the weekly home visits include: What is baby-CIMT? What is known about the effects of the method? Why is early training important? What is known about the development of hand function in children with unilateral CP?

#### Baby-massage

At the start of the first treatment period, parents in the baby-massage group will receive a three-session individualised instructional course from a certified baby-massage instructor. The sessions will be held once weekly for the first 3 weeks of the period. During the course, the parents will receive verbal and hands-on training about the purpose and mode of the massage, practising the technique on their infants under instructor supervision. The programme will cover full-body massage using a small amount of massage oil. Parents will be taught to massage each body part in sequence using slow and gentle strokes, smooth circular movements, and gentle squeezing depending on the body part. An instruction sheet will be given to parents, who will practice the technique on an ongoing basis at home.

For the daily practice, parents are instructed to choose a time of day when the infant is calm and the parents feel relaxed. A full programme takes about 30 minutes, but a partial session can be administered depending on the infant’s mood. Baby-massage is only successful if both parents and infant are enjoying the situation and must be stopped if the infant displays any distress at the practice. The massage times/durations will be noted in a diary.

### Outcome measure and procedure

The study timeline is presented in Figure 1. The outcome measure will be determined at the start of the intervention, 12 months after the intervention, and at 2 years of age. The infants will be monitored using the Hand Assessment for Infants (HAI) tool, the Alberta Infant Motor Scale (AIMS), and the Parenting Sense of Competence Scale (PSCS). Additional HAI data will be collected after the first treatment period of 6 weeks of training, after the 6 weeks of no training, after the second treatment period of 6 weeks of training and at 12 months. AHA will be administered at 2 years of age.

#### Primary outcome measure is the Hand Assessment for Infants, HAI

HAI, which is currently being developed by Krumlinde-Sundholm et al. [24,44], is intended to evaluate the quality of goal-directed manual actions in infants, 3–12 months of age, at risk of developing unilateral CP. The test procedure comprises a semi-structured video-recorded 10–15-min play session. The child is seated in a baby seat/bouncer or a high chair depending on his or her age. The chair should not restrict arm movements and as upright a position as possible should be striven for. A test kit of carefully selected toys will be presented to the infant to encourage and elicit exploration, making a wide range of motor actions observable. The set-up and administration of the play session are crucial for the possibility of observing and scoring the infant’s manual abilities. Scoring is performed from the video-recording. HAI is intended to detect and quantify possible asymmetry between hands by providing scores for each hand separately, and to provide a measure of bilateral hand use. Both criterion- and norm-referenced outcome measures will be provided. HAI is still under development, but the preliminary scale consists of 18 items (13 unimanual and 5 bimanual) each scored using a 3-point rating scale. Preliminary Rasch analysis indicates promising results in terms of internal construct validity and unidimensionality.

#### Secondary assessment

##### Questionnaire about parent’s experience of treatment

The parents will be asked about their experience of the interventions. These questions will address the feasibility of the intervention programme and the family’s impression of its effects; the responses will be recorded using a 4-point scale.

##### Parenting sense of competence scale, PSOC

The PSOC scale measures parents’ sense of confidence and satisfaction using a self-reported questionnaire [45,46]. Both mothers and fathers will be asked to complete the questionnaire. PSOC contains 16 statements to which the parents can respond in a six level Likert type scale, ranging from agreement to disagreement. The questionnaire yields two subscales: *skills* captures parental self-perceptions of skill and knowledge regarding parental functions, while *valuing* captures feelings of satisfaction, frustration, and interest associated with parenting. Satisfactory psychometric properties were reported by the original authors [46] and more recently by Gilmore and Cuskelly [47].

##### Alberta Infant Motor Scale, AIMS

AIMS identifies delayed or deviant motor development. It assesses the gross motor performance of infants relative to a norm-referenced sample aged 0–18 months [48,49]. It is an observational assessment focusing on milestones and the quality of posture and movement. It includes 58 items regarding prone, supine, sitting, and standing positions and the results are reported as a composite score. AIMS has good psychometric properties [48,49] and has been specially investigated for application to preterm babies [50].

##### Neuroimaging

Brain lesion characteristics will be investigated using neuroimaging scans acquired for clinical purposes. The age at imaging can vary, but we would mainly use scans performed after the age of 6 months. Conventional structural Magnetic resonance imaging (MRI) will be acquired using various imaging protocols and equipment. A 3.0 T MRI system will be used in the Astrid Lindgren Children’s Hospital. All images will be visually reassessed specifically for this study by experienced neuroradiologists unaware of the infants’ clinical diagnosis and functional outcome. The analytical protocol was developed in our group and has been applied in previous studies [51] using the primary patterns of abnormality defined and described by Ashwal et al. [52]. The basic patterns of damage will be classified as normal, white-matter damage of immaturity (WMDI), focal ischaemic or haemorrhagic lesions, brain malformations, diffuse encephalopathy infection, and miscellaneous or unclassifiable lesions.

##### Assisting Hand Assessment, AHA

The AHA, for children aged 18 months to 12 years, examines how effectively the children use their affected hand in bimanual activities [53]. A 15-minute play session is video recorded, and then 22 items are scored on a 4-point rating scale (AHA version 4.4). The total raw score is converted to an interval scale of AHA units ranging from 0 to 100 [54] where a higher measure indicates higher ability.

### Statistical methods

Analyses will be conducted on an intention-to-treat basis. Data for each assessment will be summarised for each treatment group and the descriptive statistics will be calculated depending on the data distribution for each assessment. HAI outcomes will collected at the times defined in the study protocol. The *primary efficacy variable* will be the mean change in the HAI score from baseline to the last post-treatment period after 18 weeks. The *secondary efficacy variable* will be the mean change in the HAI score from baseline to the first post-treatment period after 6 weeks and from baseline to the age of 1 year. The mean change in HAI score will be analysed using ANCOVA, including the baseline as covariate and group as a fixed factor in the model. Another efficacy variable will be the proportions of infants with improved HAI scores from baseline to the first post-treatment period after 6 weeks, to the end of the second treatment period after 18 weeks, and to the age of 1 year. The proportions will be analysed using logistic regression including baseline as covariate and group as a fixed factor. An odds ratio of > 1 will be interpreted as indicating an increased chance of improved HAI score. A significance level of 0.05 will be used.

## Discussion

This paper outlines the background and design of an RCT with two treatment groups comparing the effects of baby-CIMT and baby-massage. To our knowledge, this is the first study directly investigating the results of specific hand training in this age group. This programme is based on various pilot data collected over several years. In the pilot data, the parents’ responses when their children were older indicated that they felt the programme was feasible. There are some methodological disadvantages to home-administered programs, as the intervention quality and content might vary between families because their situations differ and cannot be controlled for. On the other hand, the advantage for the families is that they do not need to go to hospital frequently and they have learned how to stimulate their child’s hand use in their home environment. In addition, the cost–benefit ratio of such home-based programmes is high, and if baby-CIMT proves to be effective, it can readily be implemented in clinical practice.

The inclusion criteria can be problematic because it is difficult to establish a diagnosis at an early age. If the symptoms are unclear during the first assessment, the infants can be assessed a second time some weeks later to clarify whether the symptoms are still apparent before the child is included in the study. The extent to which asymmetric symptoms may spontaneously disappear at this age is not known, but before inclusion, we will confirm that the parents are observing the same symptoms as we are. If the symptoms disappear, we will not have caused harm and the parents will be reassured about their child. The diagnosis of unilateral CP will be confirmed or discounted at a later age by the child neurologist. We have considered the possibility of CIMT harming the development of the non-involved hand. Although we have not found any interruption to the development of the non-involved hand through our pilot work, we will continue to monitor this matter.

If the study hypotheses are confirmed, this project will be of significant value. Unilateral CP causes limitations that remain throughout the whole life, impacting individual autonomy and the individual’s participation in society. Even small functional improvements may be of great importance to the individual.

## Abbreviations

AHA: Assisting Hand Assessment; AIMS: Alberta Infant Motor Scale; Baby-CIMT: Baby constraint-induced movement therapy; CA: Corrected age; CP: Cerebral palsy; CIMT: Constraint induced movement therapy; HAI: Hand Assessment for Infants; MRI: Magnetic resonance imaging; PSOC: Parenting Sense of Competence scale; RCT: Randomised controlled trial; WMDI: White matter damage of immaturity.

## Competing interests

The authors, Lena Sjöstrand, Linda Ek and Kristina Tedroff, declare that they have no competing interests. Lena Krumlinde-Sundholm and Ann-Christin Eliasson are stockholders in Handfast AB a company for educational purpose. LKS is working as AHA teacher.

## Authors’ contributions

ACE conceived the study and initiated the study design together with LKS, while LS helped with implementation. ACE and LS will individually design the therapy content. ACE, LS, and KT will be responsible for recruiting patients. LS will be responsible for data collection. LE will conduct the primary statistical analysis. KT will be responsible for the neurological examination. ACE wrote the manuscript, which was critically revised by the other authors. All authors helped refine the study protocol and approved the final manuscript. The Karolinska Institutet is the grant holder.

## Pre-publication history

The pre-publication history for this paper can be accessed here:

http://www.biomedcentral.com/1471-2431/14/141/prepub
